# Characterization of the Seed Biopriming, Plant Growth-Promoting and Salinity-Ameliorating Potential of Halophilic Fungi Isolated from Hypersaline Habitats

**DOI:** 10.3390/ijms24054904

**Published:** 2023-03-03

**Authors:** Muhammad Aizaz, Waqar Ahmad, Sajjad Asaf, Ibrahim Khan, Syed Saad Jan, Safiya Salim Alamri, Saqib Bilal, Rahmatullah Jan, Kyung-Min Kim, Ahmed Al-Harrasi

**Affiliations:** 1 Natural and Medical Science Research Center, University of Nizwa, Nizwa 616, Oman; 2 Department of Engineering Technology, University of Houston, Sugar Land, TX 77479, USA; 3 Department of Applied Biosciences, Kyungpook National University, Daegu 41566, Republic of Korea

**Keywords:** halophilic fungi, salinity, plant growth promotion, hormones, identification

## Abstract

Salinity stress is one of the major abiotic factors limiting crop yield in arid and semi-arid regions. Plant growth-promoting fungi can help plants thrive in stressful conditions. In this study, we isolated and characterized 26 halophilic fungi (endophytic, rhizospheric, and soil) from the coastal region of Muscat, Oman, for plant growth-promoting activities. About 16 out of 26 fungi were found to produce IAA, and about 11 isolates (MGRF1, MGRF2, GREF1, GREF2, TQRF4, TQRF5, TQRF5, TQRF6, TQRF7, TQRF8, TQRF2) out of 26 strains were found to significantly improve seed germination and seedling growth of wheat. To evaluate the effect of the above-selected strains on salt tolerance in wheat, we grew wheat seedlings in 150 mM, 300 mM NaCl and SW (100% seawater) treatments and inoculated them with the above strains. Our findings showed that fungal strains MGRF1, MGRF2, GREF2, and TQRF9 alleviate 150 mM salt stress and increase shoot length compared to their respective control plants. However, in 300 mM stressed plants, GREF1 and TQRF9 were observed to improve shoot length. Two strains, GREF2 and TQRF8, also promoted plant growth and reduced salt stress in SW-treated plants. Like shoot length, an analogous pattern was observed in root length, and different salt stressors such as 150 mM, 300 mM, and SW reduced root length by up to 4%, 7.5%, and 19.5%, respectively. Three strains, GREF1, TQRF7, and MGRF1, had higher catalase (CAT) levels, and similar results were observed in polyphenol oxidase (PPO), and GREF1 inoculation dramatically raised the PPO level in 150 mM salt stress. The fungal strains had varying effects, with some, such as GREF1, GREF2, and TQRF9, showing a significant increase in protein content as compared to their respective control plants. Under salinity stress, the expression of *DREB2* and *DREB6* genes was reduced. However, the *WDREB2* gene, on the other hand, was shown to be highly elevated during salt stress conditions, whereas the opposite was observed in inoculated plants.

## 1. Introduction

The world population is currently around 7.8 billion and is expected to increase to 9.7 billion by 2050. Food product demand increases as the world population grows [1]. To supply the required demand for food crop productivity per unit of area planted is inequitable. The primary causes of the decreasing agricultural production are climate change, soil structure, nutrient degradation, drought, and soil salinity [2]. According to the Food and Agricultural Organization (FAO), by 2050, 50% of the world’s land mass will disappear. Salinity, which can be found in irrigated and unirrigated areas of the planet, is one of the main abiotic stresses. There is a plethora of studies on salinity’s harmful impacts on plant growth and productivity, especially in crop plants [3]. Due to drought and salt stress, plants have developed a variety of defense mechanisms against the harmful effects of NaCl and low soil water potential [4]. Salt-sensitive plants generally cannot tolerate high levels of NaCl, especially in soil [5], which results in reduced germination, growth, and biomass [6]. Increased salt levels in soil have detrimental effects on plant development and metabolism [7]. Several methods have been used to address the issue of soil acidity and salinity [8]. Wheat is an essential food crop that ranks first in the world grain production. It feeds more than 36% of the world’s population and supplies 20% of the calories and 55% of the carbohydrates [9]. Moreover, wheat is a rich source of micro and macronutrients, which are vital for human life [10]. Wheat’s physiological and biochemical activities are impacted by salinity, which has the most adverse effects on the quality and production of wheat [11].

Some environmentally friendly procedures, such as phytoremediation and bioremediation, have been employed to rehabilitate salt-affected soils [12]. Halophytes are naturally salt-tolerant plants that have evolved to thrive in saline soils [13]. There are certain halophytes that have been created or that may be used as agricultural crops [14]. Additionally, little is known about the possible role of microbes related to plants in the soil and on plant surfaces. The soil microbial community engages in various self-regulation activities [15]. Microorganisms can produce quorum-sensing molecules to communicate when circumstances require a collective physiological shift [16]. Microbial organisms and endophytes located in plant tissues can considerably aid plants in adapting to challenging environmental situations [17]. Ecologists are particularly interested in comprehending the function of microbial communities and their interactions with crops throughout their improvement and exposure to harsh environments in dry regions [18].

It has been demonstrated that microorganisms that predominately inhabit the host plant contribute to the movement of secondary metabolites, the secretion of nutrient-rich minerals, and the reduction in biotic and abiotic stresses [19]. They are effective plant growth-promoting fungi and secrete the hormones indole-3-acetic acid (IAA), and gibberellic acid (GA), which may help enhance plant growth and development [20]. It has been observed that a variety of phytohormone-producing fungi promote plant development under salinity stress conditions [21]. Previously, various fungi have been reported, such as *Trichoderma harzianum* [22] and *Penicillium* sp. [23], *Bipolaris* sp. [24], *Curvularia lunata* [25], *Aspergillus niger* [26], *Trichoderma virens* [27] that showed a positive effect on plant growth during biotic and abiotic stress conditions.

The rhizosphere’s colonization has been linked to dramatic alterations in hormone balance, and phytohormones act as messengers, governing plant development, root and shoot architecture, and secondary metabolite synthesis [28,29,30]. Auxins are phytohormones that control the growth of shoot and root meristems [31]. By synthesizing auxins, phyto-friendly soil microorganisms can directly impact plant auxin metabolism [32], or indirectly by altering endogenous plant auxin levels [33]. Previous studies suggest that the stimulating impact of certain plant growth-promoting bacteria and fungi on host plants may be primarily attributed to auxin synthesis [34]. Endophytic fungi can enhance plant growth, increase nutrient uptake and tolerance to salinity stress by producing enzymes that break down complex organic compounds, solubilizing inorganic phosphorus, and producing compounds that can alleviate the toxic effects of salt on plants [35]. Additionally, endophytic fungi can also produce compounds that can enhance plant tolerance to salt stress by modulating the plant’s stress response and regulating ion uptake and transport. By colonizing the plant’s internal tissues, endophytic fungi can help plants to grow and thrive in saline environments [36]. Soil fungi significantly support plants’ development and ability to withstand salt stress. It can also improve soil fertility by releasing nutrients, dissolving organic matter, and producing compounds that promote plant growth. In addition, soil fungi can also help plants to tolerate salt stress by regulating water uptake and distribution, modifying root morphology, and modulating the plant’s stress response. Some soil fungi, such as arbuscular mycorrhizal fungi (AMF), form symbiotic relationships with plant roots and can enhance plant growth and tolerance to salinity stress by improving nutrient uptake and water uptake [24]. For example, AMF can increase root hair development, which improves the plant’s ability to absorb water and nutrients from the soil. Additionally, some soil fungi can produce compounds that can alleviate the toxic effects of salt on plants. These compounds can protect the plant’s cells from damage and help to regulate ion uptake and transport, which is critical for maintaining plant health under salt stress [37]. Some plant growth-promoting fungi (PGPF) can promote root growth by influencing the level of endogenous plant auxin [38]. Despite the advantages of using plant-promoting fungi in advanced agricultural practices and significant progress in attempting to portray the complexity of plant-microbe interactions, there are numerous unanswered questions about how microbial assembly is created, evolved, and retained throughout the plant’s life cycle. Omics sciences have aided our understanding of plant–microbial community interactions, specifically how plants and microbes interact intimately during salinity and how microbes influence plant development, fitness, health, and adaptation to environmental perturbations such as salt stress [39,40].

In this study, we isolated and characterized endophytic, rhizospheric, and soil fungi from different sources in coastal regions in Muscat, Oman. Our objectives were to identify salt-tolerant fungi with plant growth-promoting characteristics and to elucidate the mechanism behind salt stress mitigation. Furthermore, we checked the plant-promoting characteristics of the selected fungi on wheat seed germination and plant growth during normal and saline conditions.

## 2. Results

### 2.1. Microbial Isolation from Saline Soils

Samples were collected from different sources in the vicinity of the coastal area of Muscat, Oman. It included seven endophytic, 14 rhizospheric, two coastal soil, and 3 inside water soil fungi, as shown in Table 1.

### 2.2. Assessment of IAA in Fungal Culture Filtrates

The IAA assessment revealed that among the 26 screened fungi, only 18 fungal species produced IAA in their culture filtrates after 7 days of incubation on PDB (Potato Dextrose Broth). This included 5 endophytic, 11 rhizospheric, and 2 soil fungal species. All of the fungi isolated from the sand inside the water did not produce IAA in fungal culture filtrates, which is shown in Table 2.

### 2.3. Effect of Isolated Microbes on Wheat Seed Germination and Seedling Length

The culture filtrates of isolated microbes were used for bio-priming in the dark for 5 h to improve wheat seed germination and seedling growth. Results showed that wheat seed germination was significantly increased by the cultural filtrates of isolated microbes (Table 3) as compared to the control (distilled water). These results revealed that inoculation with 11 isolates (MGRF1, MGRF2, GREF1, GREF2, TQRF4, TQRF5, TQRF5, TQRF6, TQRF7, TQRF8, TQRF2) significantly increased seed germination. The results showed that after 24 h, two endophytic fungi from grass (GREF1 and GREF2) and two rhizospheric fungi from *T. Qatarensis* plant (TQRF6 and TQRF8) significantly increased germination percentages up to 60%, 80%, 80%, and 80%, respectively, as compared to control. A similar trend was observed after 48 and 72 h, where microbial inoculation significantly enhanced wheat seeds’ germination percentage. More interestingly, after 72 h, almost 100% of seeds germinated in MGRF1, TQRF6, and TQRF9 treated seeds. While 93.3% of seeds were germinated in GREF1 and TQRF7 treated plants as compared to control, where only 53.3% of seeds germinated. Some microbes, such as TQRF1, WSF1, WSF2, and SAEF1, were inhibitory, and no germination was observed in seeds inoculated with these fungi (Table 3).

### 2.4. Effect of Halophilic Fungi on Wheat Seedling Growth and Biomass

**Figure 1:**
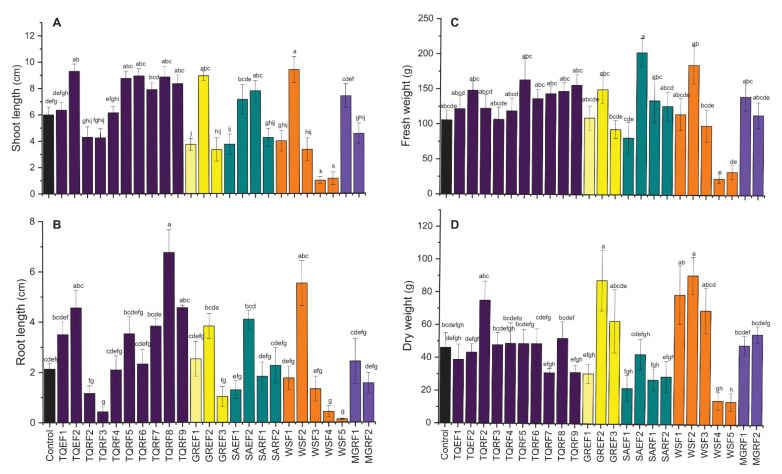
Effect of isolated fungal strains on wheat (**A**) shoot length, (**B**) root length, (**C**) fresh weight and (**D**) dry weight. Data points are the mean of three technical replications and error bars represent standard error. Bars with different letters are significantly different from each other, as evaluated by DMRT analysis.

The effect of 26 isolated fungal inoculations on wheat seedlings’ length and vigor were observed (Figure 1). Like germination percentage, these isolated fungi significantly affected the shoot and root length of wheat compared to the control. The results revealed that 12 fungi out of these 26 positively affect the shoot length of wheat. The highest shoot length was observed in GREF2, TQEF2, WSF2, TQRF6, TQRF5, TQRF8, and TQRF9 treated plants as compared to non-treated control plants. Interesting results were observed with endophytic fungi, which significantly reduced the shoot length of wheat compared to the control (Figure 1A–D).

Furthermore, shoot length was highly reduced in WSF4, and WSF5 treated plants. On the other hand, in root length, a similar trend was observed, and some of the fungi isolated from different sources showed promotory results. A significant increase was observed in the root length of wheat treated with TQEF1, SAEF2, TQRF2, WSF2, TQRF8, TQRF9, and GREF2 as compared to the control. The longest root length was observed in TQRF8, followed by WSF2 and TQEF2, as compared to other isolates and control. Similarly, some of the fungi were found to reduce the root length of wheat as compared to controls such as TQRF3, WSF4, and WSF5. We also observed the fresh weight of plants and found that fungal-treated seeds showed higher biomass compared to the control. Most of the fungal strains, whether they increased shoot length or root length, have been found to increase seedling biomass. A considerable increase was observed in SAEF2 and WSF2-treated plants as compared to control and other treatments. Overall, based on these results, we selected a total of eight strains for further experiments and used these strains for plant growth promotion during high salt stress conditions.

### 2.5. Effect of Fungal Cultural Filtrates on Wheat Plants Growth under Salinity Stress

**Figure 2:**
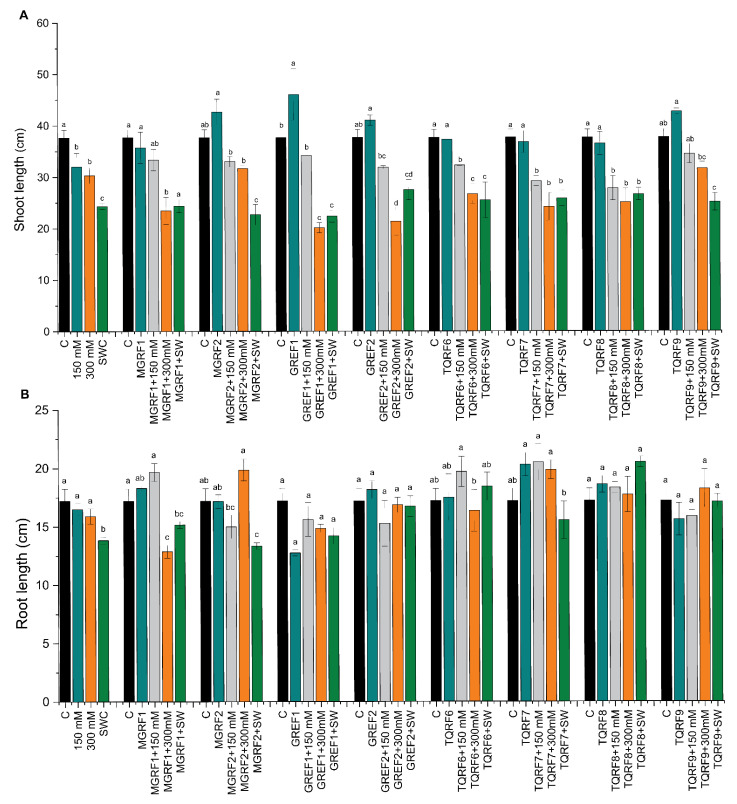
Effects of salt stress (150 mM, 300 mM, and seawater 100%) and microbial inoculation on wheat: (**A**) shoot length; and (**B**) root length. Data points are the mean of three technical replications, and error bars represent standard error. Bars with different letters are significantly different from each other, as evaluated by DMRT analysis.

Based on previous experiments, we selected eight fungal strains (MGRF1, MGRF2, GREF1, GREF2, TQRF6, TQRF7, TQRF8, and TQRF9) for their growth promotory role in wheat plants. Our results revealed that plant growth exposed to salt stress was significantly reduced as compared to the control (Figure 2A,B). This growth reduction was directly proportioned to salt concentration, and 150 mM, 300 mM, and SW reduced the shoot length by 15%, 19%, and 35%, and root length by 4%, 7.5%, and 19.5%, compared to control plants (Figure 2A,B). Data reveals that these fungal strains showed various effects on wheat shoot length compared to the control. A significant increase of up to 22% was found in GREF1-inoculated plants. Our results revealed that fungal strains MGRF1, MGRF2, GREF2, and TQRF9 mitigate 150 mM salt stress and increase shoot length as compared to their respective control plants. While in the case of 300 mM stressed plants, GREF1 and TQRF9 were found to promote shoot length compared to their control plants. However, the above results show that SW (seawater 100%) reduced the shoot length to 35% compared to the control (Figure 2A,B). Some fungal strains, such as GREF2 and TQRF8, showed promotory effects and mitigated salt stress in SW-treated plants. Like shoot length, a similar trend was observed in root length, and different salt stresses such as 150 mM, 300 mM, and SW reduced root length to 4%, 7.5%, and 19.5%, respectively. However, on the other hand, the selected fungal strains showed a significant increase in the root length of wheat plants in 150 mM, 300 mM, and SW stress conditions. Similarly, TQRF7, MGRF1, and GREF2 were found to significantly increase root length in 150 mM stress as compared to their respective control plants. In general, data show that TQRF7 significantly increased the root length by 18%, 19.1%, and 15.5%, under 150 mM and 300 mM salt compared to the control. We also noted that TQRF8 significantly increased (19.5%) the root length in SW.

**Figure 3:**
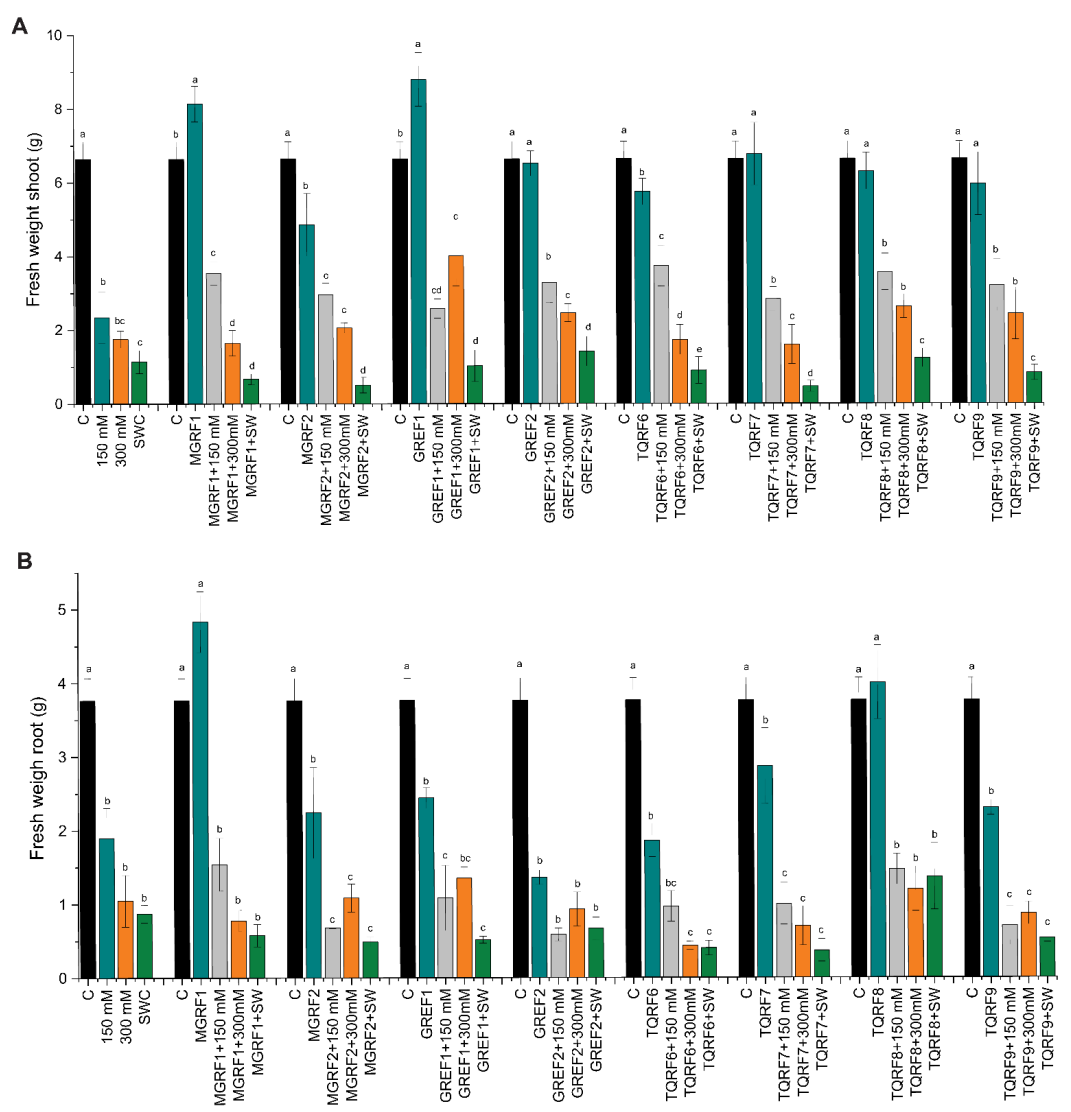
Effects of salt stress (150 mM, 300 mM, and seawater 100%) and microbial inoculation on wheat: (**A**) shoot fresh weight; and (**B**) root fresh weight. Data points are the mean of three technical replications, and error bars represent standard error. Bars with different letters are significantly different from each other, as evaluated by DMRT analysis.

Salt concentration also showed an effect on the shoot and fresh root weight, and shoot fresh weight decreased to 64%, 73.5%, and 82.8% in salt concentrations of 150 mM, 300 mM, and SW, respectively, as compared to the control (Figure 3A,B). Similar results were observed in fresh root weight, with about 60.1%, 78%, and 81.7% reductions observed in 150 mM, 300 mM, and SW salt stress as compared to control plants. We observed that plants inoculated with fungal strains significantly increase shoot and root fresh weight and mitigate salt stress. A significant increase was observed in almost all strains during 150 mM stress conditions. However, during SW stress, only GREF2 showed a positive effect and increased fresh weight as compared to the control.

### 2.6. Regulation of Enzymatic and Non-Enzymatic Antioxidants under Salinity Stress

**Figure 4:**
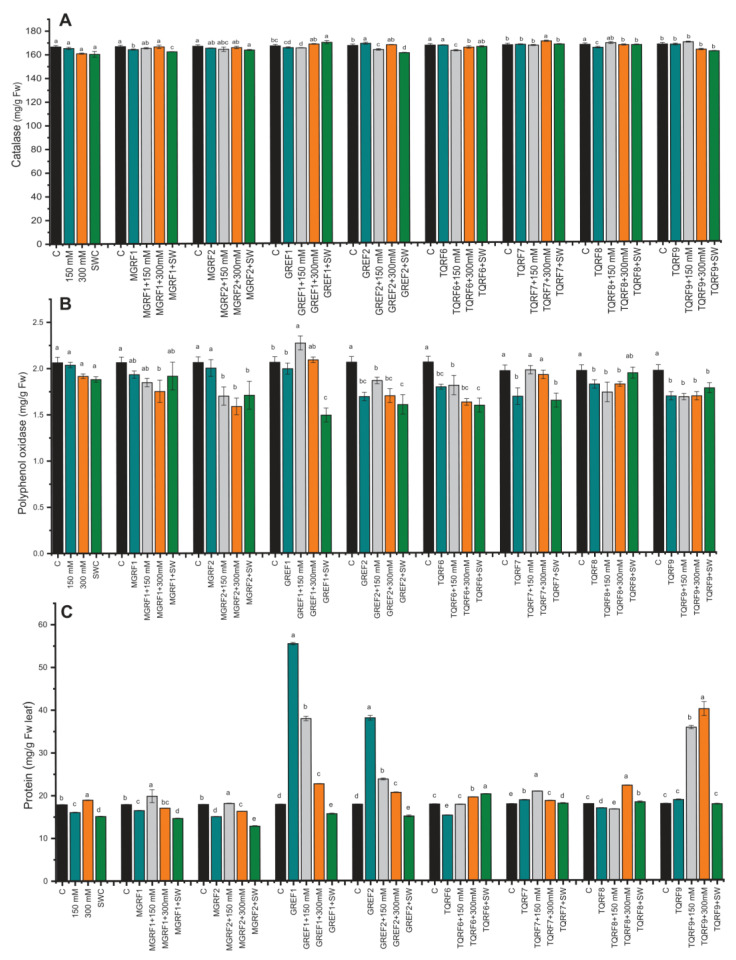
Effects of isolated fungal strains on wheat antioxidant content during salt stress (150 mM, 300 mM, and seawater 100%): (**A**) Catalase (CAT); (**B**) Polyphenol oxidase (PPO); and (**C**) Total protein (TP) contents. Data points are the mean of three technical replications, and error bars represent standard error. Bars with different letters are significantly different from each other, as evaluated by DMRT analysis.

Different enzymatic and non-enzymatic antioxidant activities were determined in wheat plants under NaCl stress and in fungal-inoculated plants. Catalase levels decreased in salt-treated plants as compared to control plants. However, some fungal strains such as GREF1, TQRF7, and MGRF1 showed an increase in CAT levels (Figure 4A). Most of the fungal strains showed a rise with 150 mM and 300 mM salt stress. Similar results were observed in polyphenol oxidase (PPO), and the GREF1 strain showed an upsurge in 150 mM salt stress compared to its respective control (Figure 4B). In total protein contents (TP), a reduction was observed in 150 mM and SW, while an increase was found in 300 mM salt stress (Figure 4C). The fungal strains showed different effects, and some strains, such as GREF1, GREF2, and TQRF9, showed significant enhancement in protein contents as compared to their respective control plants. Inoculation of GREF1 and GREF2 significantly (210.1% and 112.8%) improved the protein contents as compared to the control. GREF1 dramatically (111.1%) enhanced the protein content under 150 mM salt concentration while most of the microbes significantly boosted the protein content in 300 mM, but notably, we observed that TQRF9 showed a 123.8% increase as compared to control and inoculated treated plants.

### 2.7. Determination of Flavonoids and Flavonols Contents

**Figure 5:**
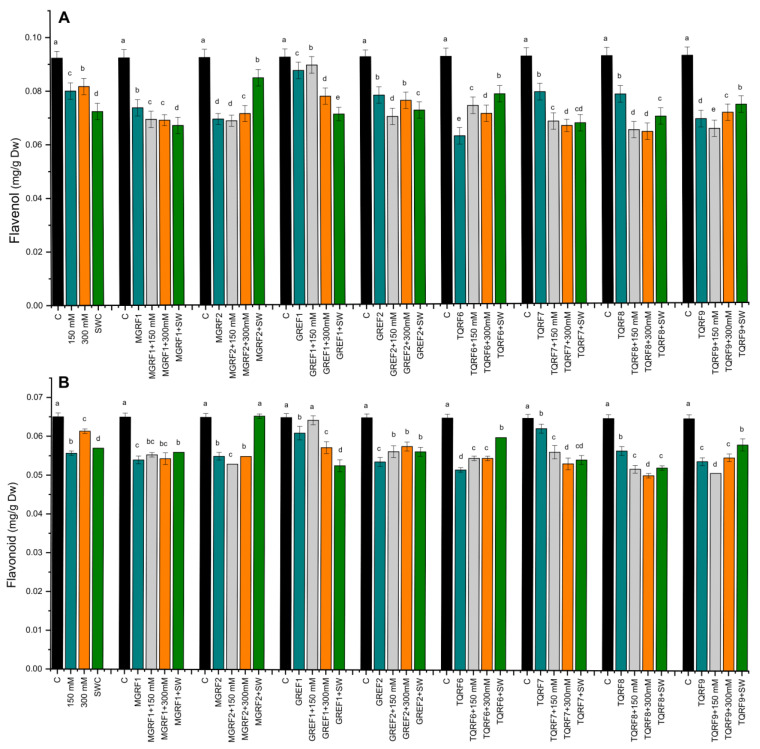
Effects of isolated fungal strains on: (**A**) Flavonol; and (**B**) Flavonoid contents of wheat plants during salt stress (150 mM, 300 mM, and seawater 100%). Data points are the mean of three technical replications and error bars represent standard error. Bars with different letters are significantly different from each other, as evaluated by DMRT analysis.

The results revealed that salt stress significantly increased flavonoid contents compared to control plants. In the case of flavonoid contents, the fungal strains did not show any considerable effect except for TQRF9 and GREF1, where a significant increase was observed in flavonoid contents as compared to their respective control plants (Figure 5). Similar results were observed in flavonol contents and salt stress, significantly inhibiting the accumulation of flavonols in wheat plants as compared to the control. Flavonols accumulation was inversely proportional to the salt concentration, and a decrease was observed in 150 mM, 300 mM, and SW, respectively (Figure 5). The fungal inoculation did not show much significant change in flavonol accumulation, except for the GREF1 strain, which showed a significant increase in 150 mM salt stress compared to respective control plants.

### 2.8. Gene Expression under Salinity Stress and Fungal Inoculation

**Figure 6:**
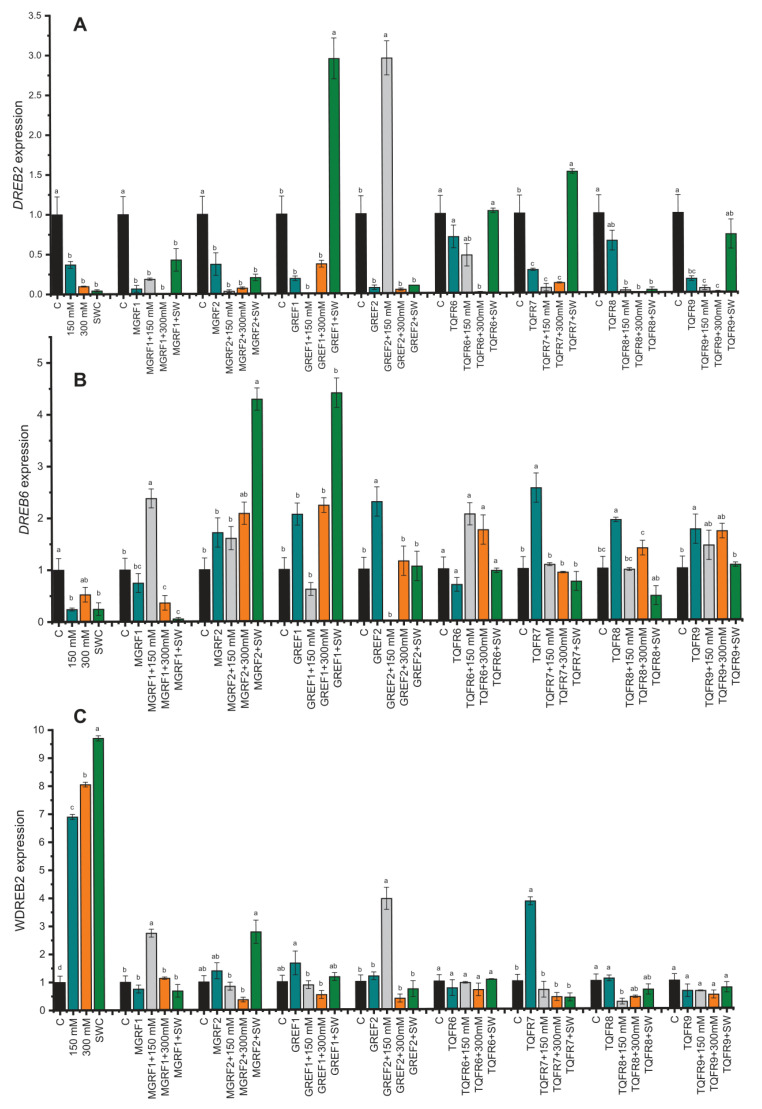
Relative expression of *WDREB2* (**A**), *DREB2 (***B)**, and *DREB6* (**C**) genes in wheat plants with and without inoculation of eight fungal strains under salt stress (150 mM, 300 mM, and seawater 100%). The values are means of three technical replicates calculated relative to those of actin gene expression. Error bars represent standard error. Bars with different letters are significantly different from each other, as evaluated by DMRT analysis.

The relative expression of genes associated with abiotic stress and microbial signaling was examined to determine the molecular mechanism behind the reduction in salt stress in wheat plants. In the current study, we evaluated *DREB2* (GU785008.1), *DREB6* (AY781361.1), and *WDREB2* (AB193608.1), by RT–qPCR (Quantitative Reverse Transcription Polymerase Chain Reaction), which are genes reported to be closely associated with osmotic stress. The expression levels of *DREB2* and *DREB6* were downregulated under salinity stress (Figure 6A). However, the *WDREB2* gene was found to be highly upregulated during salt stress conditions which was directly proportional to salt concentration. GREF1 and GREF2 inoculated plants showed increased expression of the *DREB2* gene in 100% of plants under 150 mM salinity stress (Figure 6). In the case of fungal-inoculated plants, *WDREB2* expression was found to be downregulated during salt stress. On the other hand, *DREB6* was suppressed with salt stress, while after fungal application, it was found to be upregulated. The results showed that the expression of *DREB6* was significantly higher in endophyte-inoculated plants than in non-inoculated control plants, especially in MGRF2- and GREF1-treated plants.

### 2.9. PCA Biplot Analysis

**Figure 7:**
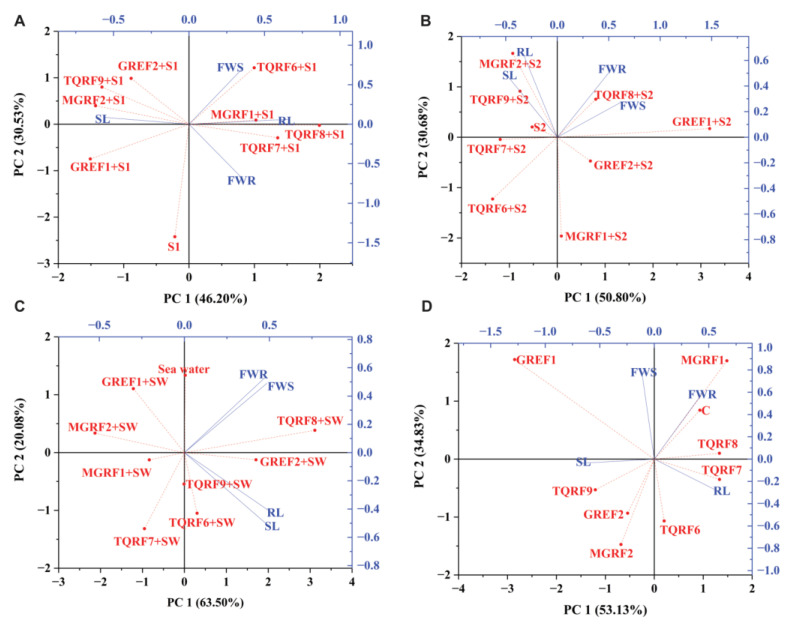
Principle Component Analysis (PCA) Biplots: (**A**) Control Biplots; (**B**) 150 mM; (**C**) 300 mM; and (**D**) 100% Sea water.

PCA had disseminated plant biomasses in all four quarters of biplots (Figure 7). The objective of PCA was to demonstrate the number of components that can be extracted to decrease the number of effective dimensions. In this study, four variables of data were collected from control, 150 mM, 300 mM, and SW salt-stressed plants. Fungal strains were used to alleviate the salinity stress and promote plant root length, shoot length, and root and shoot fresh weight in the principal component analysis. In PCA plots, PC1 was greater than PC2, while the eigenvalue increased or decreased by one but induced the cumulative value under salinity stress (Figure 7). At control, 150 mM, 300 mM, and SW, stress conditions were recognized in biplots at 51.63%, 36.48%, 49.95%, and 54.71% cumulative values (Table 4). The plant’s biomasses were constructed on factor score and analyses for thirteen inoculated wheat plants. The PCA result showed that four variables at control, S1 (150 mM), S2 (300 mM), and SW (Sea water 100%), determined the inoculated TQRF8 and TQRF7 most resistant in salinity stress. Furthermore, the TQRF7 and TQRF8 biplots were similarly found at control and 150 mM. With 300 mM and 100% salinity stress, TQRF3 and TQRF8 were contrasted on the biplot.

### 2.10. Fungal Strain Identification and Phylogenetic Analysis

**Figure 8:**
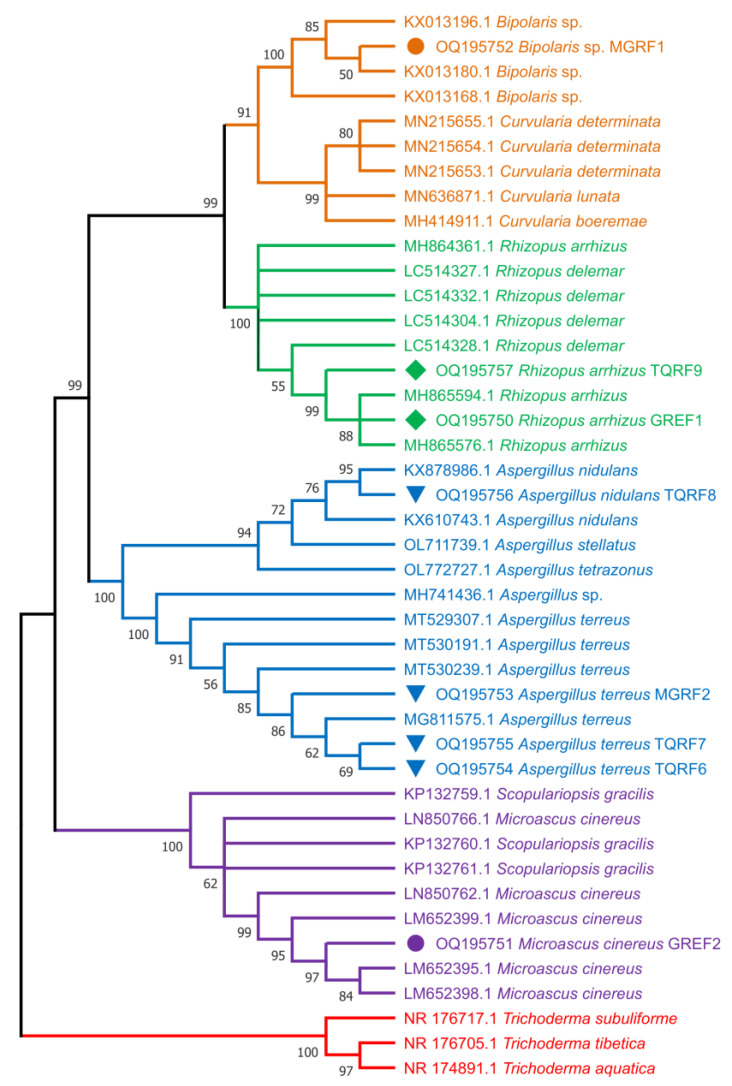
Molecular phylogenetic analysis of eight fungal strains used in this study from ITS region using the neighbor joining (NJ) method.

To identify the selected eight fungal (MGRF1, MGRF2, GREF1, GREF2, TQRF6, TQRF7, TQRF8, and TQRF9) isolates and to infer their phylogenetic position, the sequenced ITS region of the isolates were compared to the sequences in the NCBI database through BLAST search analysis (http://www.ncbi.nlm.nih.gov/, accessed on 25 January 2023). The results revealed that MGRF1 exhibited a higher level of ITS sequence identity to *Bipolaris* sp., MGRF2, TQRF6, and TQRF7 showed similarity with *Aspergillus terreus*, GREF1, and TQRF9 showed similarity with *Rhizopus arrhizus*, TQRF8 and GREF2 showed similarity with *Aspergillus nidulans* and *Microascus cinereus*, respectively. The neighbor joining (NJ) method was employed to construct a phylogenetic tree for ITS with MEGA 6 after sequence alignment with Clustal W (version 7.222), keeping default parameters. The results revealed that on the basis of ITS regions, MGRF1 formed a single clade with *Bipolaris* species, while MGRF2, TQRF6, and TQRF7 grouped with *Aspergillus terreus*, GREF1 and TQRF9 formed a clade with *Rhizopus arrhizus* supported by a relatively strong bootstrap value (Figure 8).

## 3. Discussion

Many microbes found in plant rhizospheres produce secondary metabolites that enhance the tolerance of plants to biotic and abiotic stresses. Fungi release secondary metabolites, particularly GAs, that significantly boost plant development. Several investigations have already revealed GAs synthesized by fungus species and their ability to enhance plant growth [41]. Many fungus species, including *Phoma* sp., *P. funiculosum*, and *Aspergillus* sp., have previously been observed to produce GAs in culture filtrates [41]. Fungi that synthesize GAs in the rhizosphere soil or within host plant roots act as an external source of GAs and IAA, which enhance plant growth. In the past, several fungal endophytes were identified using the 18S internal transcribed spacer (ITS) [36,42]. The fungal strains used in this study were identified based on morphological characteristics, and sequences of ITS regions. The phylogenetic analysis using the neighbor joining (NJ) method identified MGRF1 as *Bipolaris* sp., MGRF2, TQRF6 and TQRF7 as *Aspergillus terreus*, GREF1 and TQRF9 as *Rhizopus arrhizus*, TQRF8 as *Aspergillus nidulans* and GREF2 as *Microascus cinereus* using ITS regions.

Salt stress inhibits crop plant growth by influencing normal morphological, physiological, and biochemical processes [43]. Previous results revealed that salinity stresses caused a significant decrease in growth parameters as compared to control plants. Numerous crops of the legume family, such as *Sulla carnosa* have shown that salt and other abiotic stressors reduce plant development and biomass [44]. Salinity stress affects primary root growth by inhibiting cell division and cell elongation [45]. However, fungal endophyte interactions alleviated the deleterious effects of salt stress on plant growth, resulting in more biomass than control plants. Previous research found that mycorrhizal fungi helped plants to resist salt stress [46]. Our findings reveal that the existence of isolated strains stimulated the growth of salt-stressed wheat plants. Similar results were reported by [47], who found an increase in plant growth characteristics of beans with the inoculation of *P. putida* and *P. fluorescens* under salinity conditions. Several reasons have been proposed to comprehend how microbes may boost plant resistance to salinity stress; one mechanism is the synthesis of growth regulators, which leads to enhanced root growth, higher nutrient uptake, and overall plant growth under salt stress conditions [48]. Fungal endophytes produced growth-stimulating secondary metabolites such GAs and IAA, resulting in increased plant development under adverse environmental conditions [21,25]. During saline conditions, wheat plants coupled with isolated fungal strains demonstrated dramatically increased growth and shoot biomass. The increase in fresh biomass of fungal-associated seedlings was attributable to enhanced dry biomass. Previous research has shown that plants coupled with endophytic, rhizospheric, and soil fungi can mitigate the deleterious effects of salinity on *Lycopersicon esculentum* (tomato) [49], *Pennisetum glaucum* (bajra) [50], *G. max* [51], *Lactuca sativa* (lettuce) [52] and citrus [53]. Previously, active colonization of *Pe. minioluteum* (endophytic fungus) in the roots of host plants before and after salt treatment has been documented [54]. According to the data, the total phenolic content rose with salinity, and the accumulation of phenolic compounds when the plant was treated with different microorganisms was substantially higher than in non-inoculated plants. A microbial strain revealed strong resistance towards salinity stress on wheat plants. Under saline conditions, seed germination and seedling growth of different crops such as *Sulla carnosa* [44], wheat [55], and rice [56] has been reported. However, plant tolerance to stressful environments is promoted by fungal interaction with a host plant, which improves plant tolerance to avoid or prevent stressful environments [57]. Previous findings indicated that fungi that promote plant development have a beneficial role in plant metabolism [58]. Furthermore, there are relatively few instances of endophytic fungus on agricultural plants [59]. Our current results show that the selected fungi significantly increased plant growth. According to similar findings reported by many studies, PGPR enhanced the development of *Arachis hypogaea*, *Triticum aestivum,* and *Chenopodium quinoa* under salt stress [60].

Plant antioxidant enzymes have been widely investigated [61]. We studied the activities of antioxidant enzymes as the presence of NaCl causes oxidative stress in plants. Researchers have found that salt stress produces reactive oxygen species (ROS) like hydrogen peroxide, hydroxyl, and superoxide, which cause considerable cell structural damage. However, a protective system of ROS-scavenging enzymes like glutathione reductase (GR), peroxidase (POD), CAT, and superoxide dismutase (SOD) are activated when under stress. Under abiotic stress, these enzymes can minimize free radical production in cells [62]. Wheat plants inoculated with the isolated fungal strains had significantly higher antioxidant enzyme activities (CAT and SOD) under salt stress than control plants; hence, fungal-inoculated plants withstood saline conditions by lowering ROS via CAT, SOD, and ascorbate peroxidase (APX) activities. Our findings corroborate those of [63], who demonstrated that endophytic fungus under salt stress reduced H_2_O_2_ production via antioxidant enzyme activity. Similarly, plants inoculated with fungal endophytes have increased antioxidant enzyme activity, allowing them to withstand salt stress [64].

The expression of several abiotic stress and transporter genes (*DREB2*, *DREB6, WDREB2*) were evaluated with quantitative RT–PCR to further understand salinity stress interactions. *DREB2* homolog in wheat displayed alternative splicing, which accumulated differently under abiotic stresses. Our data, similar to those previously, demonstrated that *DREB2* [65] and *DREB6* [66] genes are upregulated in response to abiotic stresses involving dehydration, such as drought, high salinity, and extreme temperature changes. We noted in the given data that *WDREB2* expressed in salinity stress and previously isolated as a DREB2 homolog, is expressed in wheat seedlings, acts as a transcription factor, and positively regulates under abiotic stresses [67]. Inoculated sea water treated plants showed a higher expression level than the control.

## 4. Materials and Methods

### 4.1. Isolation of Rhizospheric, Endophytic and Soil Fungi

Endophytic and rhizospheric fungi were isolated from four plants growing near the shore of Muscat, Oman: *Paspalum vaginatum* (GR), *Tetraena qatarensis* (TQ), *Sueda Aegyptiaca* (SA), and *Avicennia marina* (MG). A total of 26 microbes were isolated from four sources (plants, rhizospheric region, soil near coastal area, and sand inside sea water). Endophytic fungi were isolated by following the protocol of [68], with minor adjustments. To eliminate dust, soil particles, and debris, the plant material was washed thoroughly with running tap water [69]. The plant material was surface sterilized by immersing it in 75% ethanol for 1 min, then in 12% sodium hypochlorite for 1 min, followed by two rinses in sterile distilled water. The plant material was dried on sterile filter paper before cutting into 3–3.5 cm sections with a sterilized blade. Using sterile forceps, four portions of each part were placed on an agar plate and cultured for six days at 26–30 °C. Fungal hyphae tips from plant tissues were subcultured on potato dextrose agar (PDA) at 26–30 °C for 10 days [70]. Subculturing fungal cultures on PDA was performed until pure isolates were obtained. The volume displacement technique was applied to isolate the fungus from the rhizosphere [71]. In beakers containing 90 mL of sterile distilled water, 2 cm pieces of roots and the adhering soil were added. Then, 10^−3^ dilutions were made and placed in flasks, which were then incubated in a shaking incubator at 25 ± 1 °C. After incubating the flasks for 15 min, the roots were lifted, the process was repeated, and new roots were added until the final volume of soil with water was 100 mL. Furthermore, samples were obtained around the roots, containing most of the microbial activity. Random soil samples were taken from the wet and dried beaches of the coastal regions and bed of seashores underwater. The collected soil samples were diluted in 10 mL of sterile distilled water with 1 g of soil [72]. On PDA plates, 1 mL of the suspension was inoculated in triplicate and incubated for 5–7 days at 28 °C.

### 4.2. Screening Fungal Strains for IAA Production

An initial assessment was performed to test the phytohormone production potential of endophytes by adding 1 mL of Salkowski reagent to 2 mL of culture filtrate. The colorimetric approach was used to screen these fungal strains for IAA synthesis [73]. The fungal strains were cultured in a shaking incubator at 120 rpm at 30 °C on Czapek–Dox broth medium. The samples were filtered after seven days, and the IAA concentrations in the culture filtrates were determined by adding 1 mL of Salkowski reagent to 2 mL of each culture filtrate, followed by 30 min of incubation in the dark. IAA production in the cultured medium was evident by a characteristic indication of reddish to pinkish color in the solution. IAA-producing strains were chosen for future study.

### 4.3. Fungi Identification and Phylogenetic Analysis

A DNeasy plant mini kit (QIAGEN, Valencia, CA, USA) was used to extract genomic DNA (gDNA) from fungal mycelia. Using the BLAST method, the fungal strains’ internal transcriber region (ITS) was amplified using PCR, analyzed, and aligned with sequences in the NCBI database. ITS1 (5′-TCCGTAGGTGAACCTGCGG-3′) and ITS2 (5′-GCTGCGTTCTTCATCGATGC-3′) fungus-specific primers were used to amplify the ITS1 region. CLUSTAL-W in MEGA 6.06 software was used to align closely related sequences. The maximum likelihood (ML) methods incorporated in MEGA X [74] were utilized to generate phylogenetic trees. Each node in the phylogenetic trees was statistically supported using 1000 bootstrap replications.

### 4.4. Seed Biopriming and Germination with Fungal Strain

Wheat seeds were obtained from the seed bank at the University of Nizwa, Oman, to be used in this experiment. Seeds were thoroughly sterilized in 2% sodium hypochlorite (Sigma Aldrich, St. Louis, MO, USA), washed four times with distilled water, and placed on filter paper to dry under sterile conditions. Furthermore, seeds were soaked in 100 mL of fungal culture suspension (10 days old) for seedling germination and vigor. Seed bio-priming was carried out in the dark for 5 h with the liquid fungal culture filtrate mentioned above at 25 °C. After priming, the seeds were carefully rinsed with distilled water and dried at room temperature on sterile filter paper for 72 h [75]. The germination test was used to determine the quality of inoculated wheat seed under ideal conditions. The sterilized filter paper was utilized as the growing medium for 20 seeds in each Petri dish (10 cm diameter). Samples were placed in a germination chamber at 25 °C, and each Petri dish received 5 mL of distilled water when needed. Germination data were collected every 24 h for up to 6 days using the below formula. After 14 days of seed germination, the length of the shoot and roots were measured with a ruler. An analytical balance was used to determine the fresh shoot and root weight of ten seedlings.

Formula % germination = No of seeds germinated/No of total seeds × 100

### 4.5. Fungal Strain’s Interaction with Wheat Plants under NaCl Stress

Wheat seeds were acquired from the KPK Agriculture Research Center in Pakistan and assessed for viability. The abovementioned technique of 2% sodium hypochlorite was used to sterilize the seeds [73]. To produce uniform plants, seeds were germinated in trays for ten days. The sterilized germination trays and pots were filled with sterile horticulture soil. The horticulture soil composition was as described by [73]. After 21 days, randomly selected uniform wheat seedlings were planted in plastic pots (10 × 9 cm) containing five plants in each pot. The experimental design included (a) Control plants (distilled water), (b) Inoculated plants with 11 fungal strains (c) Plants treated with 15 mL of 150 mM (NaCl), 300 mM (NaCl), and SW (sea water) salt treatments, (d) plants treated with 15 mL combination of 150 mM (NaCl), 300 mM (NaCl) and SW (seawater 100%) treatment and fungal strains. The growth chamber conditions were as follows: day/night cycle 14 h at 28 °C/10 h at 25 °C and 60–70% relative humidity. In the current experiments, we used seawater (100%; obtained from the beach of Muscat, Oman) and two NaCl dilutions (150 mM and 300 mM) to irrigate these plants. Fungal culture filtrate and mycelium were given three times to ensure adequate infection during the initial treatment at the time of transplanting and twice more at 1-week intervals. Plant growth characteristics that were evaluated included shoot, root length, and fresh biomass during harvest. The harvested wheat plants were promptly stored in liquid nitrogen for RNA extraction and antioxidant analysis, then kept at −80 °C for later use.

### 4.6. Determination of Protein and Catalase Activity

Protein contents were determined by the Bradford assay [76]. Fresh leaf ground in liquid nitrogen, used 1 mL sodium phosphate buffer (100 mM, PH 7) including ethylene diamine tetra acetic acid (EDTA: 1 mM), MgCl2 (3 mM), PVP (2%), and Tris 50 mM and 10 min centrifugation at (10,000× *g*) to obtain the resultant crude mixture (150 μL supernatant, 150 μL distilled water and 300 μL Bradford reagent). The protein contents were calculated at an absorption value of 595 nm using a spectrophotometer. The catalase activity was determined by the protocol developed by [77]. In brief, the sample extract was quickly combined with (0.2 M) H_2_O_2_ in 10 mM calcium phosphate buffer (PH 7.0) and analyzed using a spectrometer at an absorbance of 240 nm.

### 4.7. Determination of Total Polyphenol, Polyphenol Oxidase, Flavonoid, and Flavonol Activity

Total polyphenolic content was extracted using the Folin–Ciocalteu reagent (Sigma-Aldrich, Darmstadt, Germany). The extract (150 μL) was mixed with 2% Na_2_CO_3_ (1.5 mL) and gently vertex for 3 min. Then, the Folin–Ciocalteu mixture was put in the tube and kept for 30 min in the dark. In order to calibrate the curve, an ethanolic solution of gallic acid with different concentrations was prepared. Using the spectrophotometer, the absorbance of the standard and samples were determined at 750 nm [78]. The PPO assay was carried out according to previous studies [79]. The PPO Assay solution contained 2 mL of 0.1 M phosphate buffer having pH 6.0, 1 mL of 0.1 M catechol, and 0.5 mL of enzyme extract. The resultant sample mixture was incubated at ambient temperature for 5 min. The reaction was stopped by adding 1 mL of a 2.5 N solution of H_2_SO_4_. It was observed that purpurogallin gave absorbance at 495 nm. Blank was obtained using the same assay mixture by adding 2.5 N H_2_SO_4_ without any further incubation. TPP and PPO activity is articulated in U/mg protein. According to the total flavonoid, methanolic extracts were assessed, giving an absorption peak at 510 nm. In order to determine the total flavonoids, catechin was taken as a standard [80]. Furthermore, flavonols were determined by macerating dried powdered roots (0.5 g) in 3 mL ethyl alcohol (80%) for 24 h at RTP. The filter paper was used to filter the resulting suspension. By combining 1 mL of aluminum chloride (2%) in ethanol (95%), a resulting solution (1 mL) was obtained. After 20 min, the optical density of the combination was estimated at 390 nm [81].

### 4.8. RNA Isolation and RT–qPCR

We randomly collected leaves from each set of wheat plants following salt stress to measure the expression levels of the DREB2, WDREB2, and DREB6 genes. The leaves were frozen in liquid nitrogen and kept at 80 °C for subsequent stress analysis. To extract RNA and construct cDNA, an optimized protocol was adopted with minor modifications [82]. Tris-HCL (0.025 M, pH: 7.5) was produced with 1% *w*/*v* SDS, 20 mM EDTA, 0.25 M sodium chloride, and 4% *w*/*v* polyvinyl pyrrolidone. The powdered plant (100 mg) was carefully transferred to 2 mL RNase-free microtubes containing extraction buffer (750 µL). Then, an equal amount of chloroform isoamyl alcohol (CI; 24:1 *v*/*v*) was blended with it. In each tube, an equal volume of PCI (phenol:chloroform:isoamyl alcohol; 25:24:1 *v*/*v*) was mixed with the supernatant. The resulting mixes were smoothly shaken and centrifuged (12,000× *g*, 4 °C for 10 min). After that, the upper (clean and clear) layer was transferred to a (1.5 mL) microcentrifuge tube, and 1/10 volume of sodium acetate (3 M, pH 5.2) was applied. Finally, all the samples were centrifuged again (12,000× *g*, 4 °C for 10 min), and the pellet was cleaned with 75% pure ethanol. After 5 min of air drying, the pellet was dissolved in 50 µL of TE buffer. The RNA was then tested using a Nano Drop and the Qubit broad range kit (3.0), and the quality was confirmed using gel electrophoresis. In PCR tubes, 10 µL (>100 ng/L) of extracted RNA and an initially prepared Master Mix (RT buffer (2 µL), 25x dNTPs (0.8 µL), random primers (2 µL), reverse transcriptase (1 µL), and nuclease-free water (3.2 µL) were used to synthesize cDNA. The PCR reaction was cycled through a thermo cycler at 25 °C for 10 min, 37 °C for 2 h, and 85 °C for 5 min, with the temperature adjusted accordingly. When the reaction was finished, the cDNA was quantified using a Qubit DNA broad-range kit and kept at −80 °C for molecular analysis. The primer sequence and accession number of each gene is shown in Table S1. The amplified cDNA was used to perform RT–qPCR to determine stress-related genes’ relative gene expression configurations. The actin gene was used as a reference gene. Power SYBR Green Master Mix and primers (forward and reverse 10 pM) were used in a thermocycler to perform PCR reactions for all genes of interest. To reduce the experimental error, the reaction was performed three times for each sample. The following PCR conditions were used: 10 min at 94 °C, followed by 35 cycles at 94 °C (45 s), 65 °C (45 s), and 72 °C (1 min), with an extension step at 72 °C (10 min). The gene amplification threshold was set at 0.1. Each sample was run three times with three different replicates.

### 4.9. Statistical Analysis

All assays were carried out in triplicate, with data from each replicate combined. Data were analyzed using analysis of variance and Duncan’s multiple range test. A completely randomized design was adopted to compare the mean values of different treatments.

## 5. Conclusions

The data of the recent study show that isolated endophytic fungi significantly increased the germination percentage and seedling growth of wheat plants during normal, as well as in salt stress conditions. Plant biomass was found to increase by using these halophilic fungi in salt-stressed environments. Plants exposed to salt had lower antioxidant activity than plants treated with salinity and growth-promoting microbes. Since it is a saline-sensitive plant, salt stress causes unfavorable morphological, physiological, and biochemical changes in wheat. In the present experiment, the augmented level of catalase and protein activity in selected microbes contributes to maintaining cellular homeostasis against salinity stress. Under stress and non-stress conditions, selected microbes significantly positively affected wheat’s biochemical and physiological parameters. The stress-adapted fungi reduce the negative impacts of salinity stress by activating the antioxidant system of the plants.

## Figures and Tables

**Table 1:** Fungi isolated from different sources in Muscat, Oman.

Isolated Microbes			
Endophytic Fungi	Rhizospheric Fungi	Soil Fungi Coastal Region	Sand Fungi Inside Water
TQEF1	TQRF1	SASF1	WSF1
TQEF2	TQRF2	TQRF4	WSF2
SAEF1	TQRF3		WSF3
SAEF2	MGRF1		
GREF1	MGRF2		
GREF2	TQRF5		
GREF3	TQRF6		
	TQRF7		
	TQRF8		
	TQRF9		
	SARF1		
	SARF2		
	WSF4		
	WSF5		

**Table 2:** Selected microbes for IAA production.

S. No	Strain	IAA Production	S. No	Strain	IAA Production
1	TQEF1	+	14	SARF1	+
2	TQEF2	−	15	SARF2	+
3	TQRF1	+	16	SASF1	+
4	TQRF2	−	17	MGRF1	−
5	TQRF3	−	18	MGRF2	+
6	TQRF4	+	19	GREF1	+
7	TQRF5	+	20	GREF2	+
8	TQRF6	+	21	GREF3	+
9	TQRF7	+	22	WSF1	−
10	TQRF8	+	23	WSF2	−
11	TQRF9	+	24	WSF3	−
12	SAEF1	−	25	WSF4	+
13	SAEF2	+	26	WSF5	+

**Table 3:** Effect of isolated fungi on wheat seeds germination percentage.

S.No	Strain	D1	D2	D3	D4	D5	D6
1	Control	0	53.3	53.3	53.3	53.3	53.3
2	TQEF1	6.6	66.6	73.3	73.3	0	73.3
3	TQEF2	13.3	0	46.6	73.3	73.3	86.6
4	TQRF1	0	0	0	0	0	0
5	TQRF2	13.3	0	26.6	26.6	26.6	26.6
6	TQRF3	6.6	0	6.6	13.3	26.6	33.3
7	TQRF4	26.6	40	60	60	66.6	73.3
8	TQRF5	6.6	33.3	66.6	73.3	100	100
9	TQRF6	80	80	100	100	100	100
10	TQRF7	46.6	66.6	93.3	93.3	100	100
11	TQRF8	80.3	80	100	100	100	100
12	TQRF9	66.6	80	100	100	100	100
13	SASF1	0	0	0	0	0	0
14	SAEF1	0	6.6	13.3	13.3	13.3	13.3
15	SAEF2	26.6	46.6	46.6	46.6	0	0
16	SARF1	13.3	13.3	13.3	13.3	13.3	13.3
17	SARF2	0	13.3	13.3	13.3	13.3	13.3
18	GREF1	60	66.6	93.3	93.3	100	100
19	GREF2	80	80	86.6	86.6	86.6	86.6
20	GREF3	6.6	0	20	20	20	26.6
21	WSF1	6.6	0	6.6	26.6	26.6	26.6
22	WSF2	13.3	0	40	66.6	66.6	66.6
23	WSF3	0	0	26.6	26.6	26.6	33.3
24	WSF4	0	0	0	0	0	6.6
25	WSF5	0	0	0	0	0	6.6
26	MGRF1	46.6	100	100	100	100	100
27	MGRF2	40	53.3	53.3	60	60	60

**Table 4:** Eigen values, variability (%), and cumulative (%) of Control, 150 mM, 300 mM, 300 mM and 100% sea water axes of PCA.

**Control**	**Eigenvalue**	**Percentage of Variance**	**Cumulative**
	2.06504	51.63%	51.63%
	1.02057	25.51%	77.14%
	0.65773	16.44%	93.58%
	0.25666	6.42%	100.00%
**150 mm**	1.45911	36.48%	36.48%
	1.09593	27.40%	63.88%
	1.01013	25.25%	89.13%
	0.43483	10.87%	100.00%
**300 mm**	1.99783	49.95%	49.95%
	1.13771	28.44%	78.39%
	0.66465	16.62%	95.00%
	0.19982	5.00%	100.00%
**100%**	2.18834	54.71%	54.71%
	0.99629	24.91%	79.62%
	0.58621	14.66%	94.27%
	0.22916	5.73%	100.00%

## Data Availability

Not applicable.

## Supplementary Materials

The supporting information can be downloaded at: https://www.mdpi.com/article/10.3390/ijms24054904/s1.

## Abbreviations

GR	Grass (*Paspalum vaginatum)*,
TQ	*Tetraena qatarensis*,
SA	*Sueda Aegyptiaca*,
MG	Mangrove (*Avicennia marina)*
WS	Water sand
S1	150 mM
S2	300 mM
SW	Sea water

## References

[1] Arti D., Choudhary M., Sourirajan A. (2020). Salt tolerant bacteria for crop improvement in saline agriculture fields: Development, challenges and opportunities. Plant Arch..

[2] Khan N., Bano A., Rahman M.A., Guo J., Kang Z., Babar M. (2019). Comparative physiological and metabolic analysis reveals a complex mechanism involved in drought tolerance in chickpea (*Cicer arietinum* L.) induced by PGPR and PGRs. Sci. Rep..

[3] de Oliveira A.B., Alencar N.L.M., Gomes-Filho E. (2013). Comparison between the water and salt stress effects on plant growth and development. Responses Org. Water Stress.

[4] Munns R., Tester M. (2008). Mechanisms of salinity tolerance. Annu. Rev. Plant Biol..

[5] Prasad S.R., Bagali P.G., Hittalmani S., Shashidhar H. (2000). Molecular mapping of quantitative trait loci associated with seedling tolerance to salt stress in rice (*Oryza sativa* L.). Curr. Sci..

[6] Neumann P.M. (2008). Coping mechanisms for crop plants in drought-prone environments. Ann. Bot..

[7] Sharma A., Dev K., Sourirajan A., Choudhary M. (2021). Isolation and characterization of salt-tolerant bacteria with plant growth-promoting activities from saline agricultural fields of Haryana, India. J. Genet. Eng. Biotechnol..

[8] Gangwar P., Singh R., Trivedi M., Tiwari R.K. (2020). Sodic soil: Management and reclamation strategies. Environmental Concerns and Sustainable Development.

[9] Hasanuzzaman M., Nahar K., Rahman A., Anee T.I., Alam M.U., Bhuiyan T.F., Oku H., Fujita M. (2017). Approaches to enhance salt stress tolerance in wheat. Wheat Improvement, Management and Utilization.

[10] World Health Organization (2019). Preventing Disease through Healthy Environments: Exposure to Highly Hazardous Pesticides: A Major Public Health Concern.

[11] Seleiman M.F., Aslam M.T., Alhammad B.A., Hassan M.U., Maqbool R., Chattha M.U., Khan I., Gitari H.I., Uslu O.S., Rana R. (2022). Salinity stress in wheat: Effects, mechanisms and management strategies. Phyton.

[12] Mishra P., Singh P.P., Singh S.K., Verma H. (2019). Sustainable agriculture and benefits of organic farming to special emphasis on PGPR. Role of Plant Growth Promoting Microorganisms in Sustainable Agriculture and Nanotechnology.

[13] Flowers T.J., Colmer T.D. (2015). Plant salt tolerance: Adaptations in halophytes. Ann. Bot..

[14] Gul B., Ansari R., Khan M.A. (2009). Salt tolerance of Salicornia utahensis from the great basin desert. Pak. J. Bot..

[15] Leach J.E., Triplett L.R., Argueso C.T., Trivedi P. (2017). Communication in the phytobiome. Cell.

[16] Chauhan H., Bagyaraj D., Selvakumar G., Sundaram S. (2015). Novel plant growth promoting rhizobacteria—Prospects and potential. Appl. Soil Ecol..

[17] Numan M., Bashir S., Khan Y., Mumtaz R., Shinwari Z.K., Khan A.L., Khan A., Ahmed A.-H. (2018). Plant growth promoting bacteria as an alternative strategy for salt tolerance in plants: A review. Microbiol. Res..

[18] Pudake R.N., Mehta C.M., Mohanta T.K., Sharma S., Varma A., Sharma A.K. (2017). Expression of four phosphate transporter genes from Finger millet (*Eleusine coracana* L.) in response to mycorrhizal colonization and Pi stress. 3 Biotech.

[19] Vandenkoornhuyse P., Quaiser A., Duhamel M., Le Van A., Dufresne A. (2015). The importance of the microbiome of the plant holobiont. New Phytol..

[20] Radhakrishnan R., Shim K.-B., Lee B.-W., Hwang C.-D., Pae S.-B., Park C.-H., Kim S.-U., Lee C.-K., Baek I.-Y. (2013). IAA-producing *Penicillium* sp. NICS01 triggers plant growth and suppresses *Fusarium* sp.-induced oxidative stress in sesame (*Sesamum indicum* L.). J. Microbiol. Biotechnol..

[21] Bilal L., Asaf S., Hamayun M., Gul H., Iqbal A., Ullah I., Lee I.-J., Hussain A. (2018). Plant growth promoting endophytic fungi Asprgillus fumigatus TS1 and Fusarium proliferatum BRL1 produce gibberellins and regulates plant endogenous hormones. Symbiosis.

[22] Mbarki S., Cerdà A., Brestic M., Mahendra R., Abdelly C., Pascual J.A. (2017). Vineyard compost supplemented with Trichoderma harzianum T78 improve saline soil quality. Land Degrad. Dev..

[23] Radhakrishnan R., Kang S.-M., Baek I.-Y., Lee I.-J. (2014). Characterization of plant growth-promoting traits of Penicillium species against the effects of high soil salinity and root disease. J. Plant Interact..

[24] Lubna, Khan M.A., Asaf S., Jan R., Waqas M., Kim K.-M., Lee I.-J. (2022). Endophytic fungus *Bipolaris* sp. CSL-1 induces salt tolerance in *Glycine max*. L via modulating its endogenous hormones, antioxidative system and gene expression. J. Plant Interact..

[25] Asaf S., Jan R., Khan M.A., Khan A.L., Asif S., Bilal S., Ahmad W., Waqas M., Kim K.-M., Ahmed A.-H. (2022). Unraveling the mutualistic interaction between endophytic Curvularia lunata CSL1 and tomato to mitigate cadmium (Cd) toxicity via transcriptomic insights. Sci. Total Environ..

[26] Asaf S., Jan R., Khan A.L., Bilal S., Asif S., Al-Harrasi A., Kim K.-M. (2022). Unraveling the Genome Sequence of Plant Growth Promoting Aspergillus niger (CSR3) Provides Insight into the Synthesis of Secondary Metabolites and Its Comparative Genomics. J. Fungi.

[27] Bilal S., Shahzad R., Asaf S., Imran M., Al-Harrasi A., Lee I.-J. (2023). Efficacy of endophytic SB10 and glycine betaine duo in alleviating phytotoxic impact of combined heat and salinity in *Glycine max* L. via regulation of redox homeostasis and physiological and molecular responses. Environ. Pollut..

[28] Dodd I., Zinovkina N., Safronova V., Belimov A. (2010). Rhizobacterial mediation of plant hormone status. Ann. Appl. Biol..

[29] Spaepen S., Bossuyt S., Engelen K., Marchal K., Vanderleyden J. (2014). Phenotypical and molecular responses of A rabidopsis thaliana roots as a result of inoculation with the auxin-producing bacterium A zospirillum brasilense. New Phytol..

[30] Verbon E.H., Liberman L.M. (2016). Beneficial microbes affect endogenous mechanisms controlling root development. Trends Plant Sci..

[31] Demeulenaere M.J., Beeckman T. (2014). The interplay between auxin and the cell cycle during plant development. Auxin and Its Role in Plant Development.

[32] Spaepen S., Vanderleyden J., Remans R. (2007). Indole-3-acetic acid in microbial and microorganism-plant signaling. FEMS Microbiol. Rev..

[33] Poupin M.J., Greve M., Carmona V., Pinedo I. (2016). A complex molecular interplay of auxin and ethylene signaling pathways is involved in Arabidopsis growth promotion by Burkholderia phytofirmans PsJN. Front. Plant Sci..

[34] Iqbal A., Hasnain S. (2013). Auxin producing Pseudomonas strains: Biological candidates to modulate the growth of Triticum aestivum beneficially. Am. J. Plant Sci..

[35] Johnson J.M., Alex T., Oelmüller R. (2014). Piriformospora indica: The versatile and multifunctional root endophytic fungus for enhanced yield and tolerance to biotic and abiotic stress in crop plants. J. Trop. Agric..

[36] Khan A.L., Hussain J., Al-Harrasi A., Al-Rawahi A., Lee I.-J. (2015). Endophytic fungi: Resource for gibberellins and crop abiotic stress resistance. Crit. Rev. Biotechnol..

[37] Abeer H., Abd_Allah E., Alqarawi A., El-Didamony G., Alwhibi M., Egamberdieva D., Ahmad P. (2014). Alleviation of adverse impact of salinity on faba bean (*Vicia faba* L.) by arbuscular mycorrhizal fungi. Pak. J. Bot.

[38] Contreras-Cornejo H.A., Macías-Rodríguez L., Cortés-Penagos C., López-Bucio J. (2009). Trichoderma virens, a plant beneficial fungus, enhances biomass production and promotes lateral root growth through an auxin-dependent mechanism in Arabidopsis. Plant Physiol..

[39] Knief C. (2014). Analysis of plant microbe interactions in the era of next generation sequencing technologies. Front. Plant Sci..

[40] Sharma M., Sudheer S., Usmani Z., Rani R., Gupta P. (2020). Deciphering the omics of plant-microbe interaction: Perspectives and new insights. Curr. Genom..

[41] Hamayun M., Khan S.A., Khan A.L., Shin J.-H., Ahmad B., Shin D.-H., Lee I.-J. (2010). Exogenous gibberellic acid reprograms soybean to higher growth and salt stress tolerance. J. Agric. Food Chem..

[42] Waqas M., Khan A.L., Kamran M., Hamayun M., Kang S.-M., Kim Y.-H., Lee I.-J. (2012). Endophytic fungi produce gibberellins and indoleacetic acid and promotes host-plant growth during stress. Molecules.

[43] Souza R.d., Ambrosini A., Passaglia L.M. (2015). Plant growth-promoting bacteria as inoculants in agricultural soils. Genet. Mol. Biol..

[44] Hmaeid N., Wali M., Mahmoud O.M.-B., Pueyo J.J., Ghnaya T., Abdelly C. (2019). Efficient rhizobacteria promote growth and alleviate NaCl-induced stress in the plant species Sulla carnosa. Appl. Soil Ecol..

[45] Sadak M.S., El-Hameid A., Asmaa R., Zaki F.S., Dawood M.G., El-Awadi M.E. (2020). Physiological and biochemical responses of soybean (*Glycine max* L.) to cysteine application under sea salt stress. Bull. Natl. Res. Cent..

[46] Porcel R., Aroca R., Ruiz-Lozano J.M. (2012). Salinity stress alleviation using arbuscular mycorrhizal fungi. A review. Agron. Sustain. Dev..

[47] Mo W., Tang W., Du Y., Jing Y., Bu Q., Lin R. (2020). PHYTOCHROME-INTERACTING FACTOR-LIKE14 and SLENDER RICE1 interaction controls seedling growth under salt stress. Plant Physiol..

[48] Metwali E.M., Abdelmoneim T.S., Bakheit M.A., Kadasa N.M. (2015). Alleviation of salinity stress in faba bean (*Vicia faba* L.) plants by inoculation with plant growth promoting rhizobacteria (PGPR). Plant Omics.

[49] Hajiboland R., Aliasgharzadeh N., Laiegh S.F., Poschenrieder C. (2010). Colonization with arbuscular mycorrhizal fungi improves salinity tolerance of tomato (*Solanum lycopersicum* L.) plants. Plant Soil.

[50] Borde M., Dudhane M., Jite P. (2011). Growth photosynthetic activity and antioxidant responses of mycorrhizal and non-mycorrhizal bajra (*Pennisetum glaucum*) crop under salinity stress condition. Crop Prot..

[51] Khan A.L., Hamayun M., Kim Y.-H., Kang S.-M., Lee I.-J. (2011). Ameliorative symbiosis of endophyte (Penicillium funiculosum LHL06) under salt stress elevated plant growth of *Glycine max* L.. Plant Physiol. Biochem..

[52] Kohler J., Hernández J.A., Caravaca F., Roldán A. (2009). Induction of antioxidant enzymes is involved in the greater effectiveness of a PGPR versus AM fungi with respect to increasing the tolerance of lettuce to severe salt stress. Environ. Exp. Bot..

[53] Wu Q., Zou Y., Liu W., Ye X., Zai H., Zhao L. (2010). Alleviation of salt stress in citrus seedlings inoculated with mycorrhiza: Changes in leaf antioxidant defense systems. Plant Soil Environ..

[54] Khan A.L., Waqas M., Hussain J., Al-Harrasi A., Hamayun M., Lee I.-J. (2015). Phytohormones enabled endophytic fungal symbiosis improve aluminum phytoextraction in tolerant *Solanum lycopersicum*: An examples of *Penicillium janthinellum* LK5 and comparison with exogenous GA3. J. Hazard. Mater..

[55] Radi A.A., Farghaly F.A., Hamada A.M. (2013). Physiological and biochemical responses of salt-tolerant and salt-sensitive wheat and bean cultivars to salinity. J. Biol. Earth Sci..

[56] Sarkar A., Ghosh P.K., Pramanik K., Mitra S., Soren T., Pandey S., Mondal M.H., Maiti T.K. (2018). A halotolerant *Enterobacter* sp. displaying ACC deaminase activity promotes rice seedling growth under salt stress. Res. Microbiol..

[57] Cheplick G.P. (2004). Recovery from drought stress in *Lolium perenne* (Poaceae): Are fungal endophytes detrimental?. Am. J. Bot..

[58] Yeon-Sik C., In-Jung L., Jong-Myeong K., Seon-Kap H., Seok-Jong S., Ho-Youn K., Hyeokjun Y., Muhammad H., Sumera K., Ung-Han Y. (2008). Plant growth promotion and Penicillium citrinum. BMC Microbiol.

[59] Waller F., Achatz B., Baltruschat H., Fodor J., Becker K., Fischer M., Heier T., Hückelhoven R., Neumann C., von Wettstein D. (2005). The endophytic fungus Piriformospora indica reprograms barley to salt-stress tolerance, disease resistance, and higher yield. Proc. Natl. Acad. Sci. USA.

[60] Alexander A., Singh V.K., Mishra A. (2020). Halotolerant PGPR Stenotrophomonas maltophilia BJ01 induces salt tolerance by modulating physiology and biochemical activities of *Arachis hypogaea*. Front. Microbiol..

[61] Hernández J.A., Aguilar A.B., Portillo B., López-Gómez E., Beneyto J.M., García-Legaz M.F. (2003). The effect of calcium on the antioxidant enzymes from salt-treated loquat and anger plants. Funct. Plant Biol..

[62] Radhakrishnan R., Kumari B.R. (2013). Protective role of pulsed magnetic field against salt stress effects in soybean organ culture. Plant Biosyst. —Int. J. Deal. All Asp. Plant Biol..

[63] Abogadallah G.M. (2011). Differential regulation of photorespiratory gene expression by moderate and severe salt and drought stress in relation to oxidative stress. Plant Sci..

[64] Ahmad P., Hashem A., Abd-Allah E.F., Alqarawi A., John R., Egamberdieva D., Gucel S. (2015). Role of Trichoderma harzianum in mitigating NaCl stress in Indian mustard (*Brassica juncea* L.) through antioxidative defense system. Front. Plant Sci..

[65] Mondini L., Nachit M.M., Pagnotta M.A. (2015). Allelic variants in durum wheat (*Triticum turgidum* L. var. durum) DREB genes conferring tolerance to abiotic stresses. Mol. Genet. Genom..

[66] Kumar R., Masthigowda M.H., Kaur A., Bhusal N., Pandey A., Kumar S., Mishra C., Singh G., Singh G.P. (2020). Identification and characterization of multiple abiotic stress tolerance genes in wheat. Mol. Biol. Rep..

[67] Kobayashi F., Ishibashi M., Takumi S. (2008). Transcriptional activation of Cor/Lea genes and increase in abiotic stress tolerance through expression of a wheat DREB2 homolog in transgenic tobacco. Transgenic Res..

[68] Petrini O., Fisher P. (1986). Fungal endophytes in *Salicornia perennis*. Trans. Br. Mycol. Soc..

[69] Sandhu S.S., Kumar S., Aharwal R.P. (2014). Isolation and identification of endophytic fungi from *Ricinus communis* Linn. and their antibacterial activity. Int. J. Res. Pharm. Chem..

[70] Gond S., Verma V., Kumar A., Kumar V., Kharwar R. (2007). Study of endophytic fungal community from different parts of *Aegle marmelos* Correae (Rutaceae) from Varanasi (India). World J. Microbiol. Biotechnol..

[71] Reyes A., Mitchell J. (1962). Growth response of several isolates of Fusarium in rhizospheres of host and nonhost plants. Phytopathology.

[72] Waksman S.A. (1927). Principles of Soil Microbiology.

[73] Lubna L., Khan M.A., Asaf S., Jan R., Waqas M., Kim K., Lee I.-J. (2020). Plant growth promoting *Bipolaris* sp. CSL-1 mitigate salinity stress in soybean via altering endogenous phytohormonal level, antioxidants and genes expression. Res. Sq.

[74] Tamura K., Stecher G., Peterson D., Filipski A., Kumar S. (2013). MEGA6: Molecular evolutionary genetics analysis version 6.0. Mol. Biol. Evol..

[75] Mitelut A.C., Popa M.E. (2011). Seed germination bioassay for toxicity evaluation of different composting biodegradable materials. Rom. Biotechnol. Lett..

[76] Bradford M.M. (1976). A rapid and sensitive method for the quantitation of microgram quantities of protein utilizing the principle of protein-dye binding. Anal. Biochem..

[77] Aebi H. (1984). Catalase in vitro. Methods in Enzymology.

[78] Haghighi T.M., Saharkhiz M.J. (2021). Phytotoxic potential of Vitex pseudo-negundo leaf and flower extracts and analysis of phenolic compounds. Biocatal. Agric. Biotechnol..

[79] Kumar K.B., Khan P.A. (1982). Peroxidase & Polyphenol Oxidase In Excised Ragi (Eleusine Coracana Cv Pr 202) Leaves During Senescence. Indian J. Exp. Biol..

[80] Taban A., Saharkhiz M.J., Kavoosi G. (2021). Development of pre-emergence herbicide based on Arabic gum-gelatin, apple pectin and savory essential oil nano-particles: A potential green alternative to metribuzin. Int. J. Biol. Macromol..

[81] Levon V., Klymenko S. (2021). Content of anthocyanins and flavonols in the fruits of *Cornus* spp.. Agrobiodiversity Improv. Nutr. Health Life Qual..

[82] Liu L., Han R., Yu N., Zhang W., Xing L., Xie D., Peng D. (2018). A method for extracting high-quality total RNA from plant rich in polysaccharides and polyphenols using Dendrobium huoshanense. PLoS ONE.

