# Supplementary material for: Characterization of the Seed Biopriming, Plant Growth-Promoting and Salinity-Ameliorating Potential of Halophilic Fungi Isolated from Hypersaline Habitats
Source: Int J Mol Sci. 2023 Mar 3;24(5):4904. doi: 10.3390/ijms24054904 (PMC10003710; doi:10.3390/ijms24054904)
Supplement: Supplementary file 1 [file ijms-24-04904-s001.zip › ijms-2182906-supplementary.pdf]

**Table S1**. Primers used in this study.

| **Gene name** | **Forword** | **Reverse** | **Acession no** |
|---|---|---|---|
| *Dreb2* | GCGTACAACACCTTGATTTCC | AAACTCAACTCACATCTAAGC | GU785008.1 |
| *Dreb6* | CGGCTGTGGCTTGGTTCATTC | CAGTTGCCCCATTAGACATC | AY781361.1 |
| *Wdreb2* | AGATGTTGCTTCTTCCTTGCC | ATGTGCTCCTTGAAATGCTTG | AB193608.1 |
